# Solid Organ Transplant–Transmitted Tuberculosis Linked to a Community Outbreak — California, 2015

**DOI:** 10.15585/mmwr.mm6630a1

**Published:** 2017-08-04

**Authors:** Alexander Kay, Pennan M. Barry, Pallavi Annambhotla, Carol Greene, Martin Cilnis, Peter Chin-Hong, Nicholas Arger, Louise McNitt, Nikole Neidlinger, Neha Shah, Sridhar V. Basavaraju, Matthew Kuehnert, Tambi Shaw

**Affiliations:** ^1^Tuberculosis Control Branch, California Department of Public Health; ^2^Division of Healthcare Quality Promotion, National Center for Emerging and Zoonotic Infectious Diseases, CDC; ^3^Division of Infectious Disease, University of California, San Francisco; ^4^Division of Pulmonary, Critical Care, Allergy and Sleep Medicine, University of California, San Francisco; ^5^Communicable Disease Programs, Contra Costa County Public Health, California; ^6^Donor Network West, San Ramon, California; ^7^Division of TB Elimination, National Center for HIV/AIDS, Viral Hepatitis, STD, and TB Prevention, CDC.

In the spring of 2015, a local health department (LHD) in county A notified the California Department of Public Health (CDPH) about three adults with close ties to one another and a congregate community site who had received diagnoses of tuberculosis (TB) disease within a 3-month period. Subsequent review revealed matching TB genotypes indicating that the cases were likely part of a chain of TB transmission. Only three TB cases in California in the preceding 2 years shared this same genotype. One of those three previous cases occurred in a lung-transplant recipient who had no identified epidemiologic links to the outbreak. CDPH, multiple LHDs, and CDC conducted an investigation and determined that the lung-transplant donor (patient 1) was epidemiologically linked to the three outbreak cases and had a tuberculin skin test (TST) conversion detected in 2012 upon reentry at a local jail. Three other solid organ recipients from this donor were identified; none had developed TB disease. This investigation suggests that review of organ donors’ medical records from high-risk environments, such as jails, might reveal additional information about TB risk. The evaluation of TB in organ recipients could include genotyping analysis (*1*) and coordination among local, state, and national partners to evaluate the potential for donor-derived TB.

## Investigation and Findings

**Organ donor.** The adult organ donor (patient 1), from county A, was admitted to the hospital following a motor vehicle crash in the fall of 2014 (Figure). A chest computed tomography (CT) scan on admission revealed diffuse nodular infiltrates consistent with pulmonary contusions, but also raised suspicion for TB. However, a TST and interferon gamma release assay (IGRA) were negative and indeterminate, respectively. Two sputum specimens were obtained, by endotracheal aspirate and one by bronchoalveolar lavage; all were negative for acid-fast bacilli (AFB) by smear and culture. Nucleic acid amplification testing (NAAT) was not performed. On the third hospital day, the patient deteriorated to neurologic death and next-of-kin consented to organ donation. As part of predonation screening, a questionnaire was administered to next-of-kin, and they did not recall TB symptoms, prior TB infection, or TB testing for the prospective donor. Follow-up CT scan performed 5 days after admission revealed resolution of the nodular infiltrates. On the seventh hospital day, six organs (heart, two lungs, liver, and two kidneys) were recovered and transplanted into four patients. The donor had immigrated to the United States approximately 8 years earlier and had been incarcerated several times with a negative TST result less than 2 years before having a positive TST result in early 2012 upon reentry to a local jail. The donor never received a diagnosis of TB disease.

**Transplant recipient.** The transplant recipient (patient 2), from county C, received the two donor lungs in the fall of 2014 (Figure). Three months after transplantation, and before identification of cases with a matching genotype based on spoligotyping (a polymerase chain reaction [PCR]–based method) and 24-locus variable-number tandem repeat of mycobacterial interspersed repetitive units (VNTR-MIRU) (a PCR method that analyzes specific regions of the genome), the recipient developed a persistent cough and fatigue. A CT scan revealed bilateral pleural effusions with a large pericardial effusion. The effusions were drained, and sputum was collected; cultures from pleural fluid, pericardium, and sputum yielded *Mycobacterium tuberculosis.* The recipient had minimal foreign travel and no epidemiologic links to other TB cases. Pretransplant TST and IGRA were negative. Initially, the recipient’s TB was thought not to be donor-derived because of the organ donor’s negative predonation TB evaluation. The recipient responded well to TB treatment.

**FIGURE:**
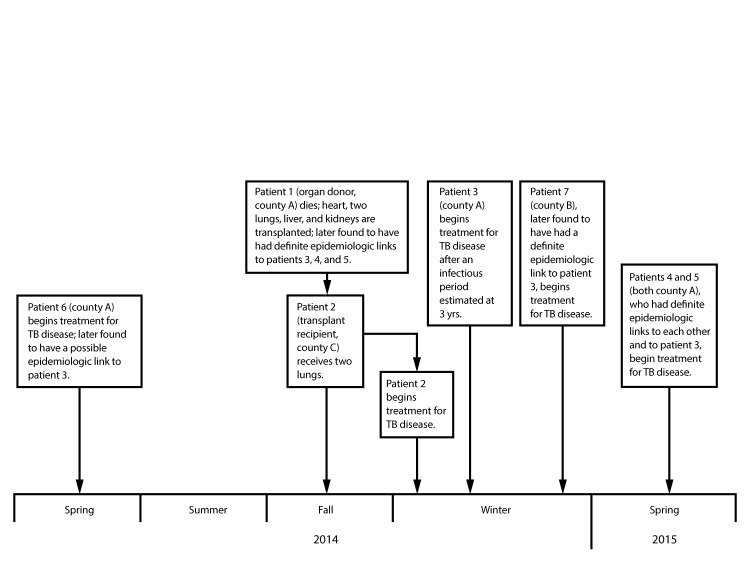
Timeline of events and epidemiologic links for a tuberculosis (TB) outbreak (N = 7*) in which an organ donor was infected with TB by one outbreak case and then transmitted TB via lung transplant to an organ recipient^†^ — California, 2014–2015 * Includes one organ donor with TB infection and six patients diagnosed with TB disease during January 2012–May 2015 with matching TB genotypes (and, if available, whole-genome sequencing results consistent with transmission), in addition to an epidemiologic link between patients. Definitions of the strength of epidemiologic links are adapted from National TB Controllers Association/CDC Advisory Group on Tuberculosis Genotyping. Guide to the application of genotyping to tuberculosis prevention and control. Atlanta, GA: US Department of Health and Human Services, CDC; 2004. ^†^ The lung-transplant donor (patient 1) had a predonation tuberculin skin test (TST) and interferon gamma release assay that were negative and indeterminate, respectively. However, investigators learned that the donor had a positive TST result upon reentry to a local jail in early 2012 after having a documented negative TST <2 years earlier during a previous incarceration.

**Outbreak.** In the spring of 2015, 3 months after TB was diagnosed in the lung transplant recipient (patient 2), county A notified CDPH about three TB cases in adults (patients 3, 4, and 5) from the same country (Figure). These patients were linked by social and familial ties, as well as a congregate community setting, and received their TB diagnoses during a 3-month period. The isolates from patients 3, 4, and 5 were subsequently found to have the same genotype, which was rare in California and the United States, and which confirmed recent transmission. Only three other TB patients in California in the preceding 2 years had this genotype (patient 2 [the transplant recipient, county C], patient 6 [county A], and patient 7 [county B]). Outbreak-associated cases for this report were defined as TB diagnoses in patients during January 2012–May 2015 with a matching genotype and an epidemiologic link (*2*). TB cases with matching genotypes not initially linked to the outbreak (patients 2, 6, and 7) were subsequently linked after reinterviews of the three patients. Reinterviews determined patients 6 and 7 had possible or definite epidemiologic links to patient 3 during patient 3’s estimated infectious period of 3 years. Subsequent whole-genome sequencing and phylogenetic analysis confirmed that the isolates from all six patients were closely related genetically. 

The lung recipient (patient 2) was reinterviewed to confirm absence of an epidemiologic link to the other patients. CDPH determined that the organ donor (patient 1) for the lung recipient had been a social contact of the three patients with outbreak-associated TB (patients 3, 4, and 5). Medical records obtained from the jail where the donor had been briefly incarcerated several times revealed documentation of a negative TST in 2010 but a positive TST (18 mm) and normal chest radiograph during incarceration in 2012. Patient 3’s lengthy estimated infectious period and the date of patient 1’s documented TST conversion from negative to positive indicate patient 3 was the most likely source case of patient 1’s TB infection. The donor did not receive therapy for latent TB. These results were not known to the organ procurement organization at the time of organ recovery.

## Public Health Response

County A and CDPH worked together to detect and prevent additional TB cases in the community. Prevention activities were intensified following identification of additional outbreak-associated cases after May 2015. CDPH also contacted the California LHDs of residence of all recipients of the donor’s organs to notify them of the potential TB risk. CDC reviewed the medical records of all of the organ recipients and contacted their treating physicians. One recipient received treatment for presumptive latent TB infection, although no further evidence of new TB infection or disease was confirmed in that patient or any other organ recipient.

## Discussion

This report describes likely donor-derived TB transmission, despite adherence to currently recommended guidelines for TB evaluation in organ donors. Investigation of the social, familial, and congregate community setting led to identification of the linkage between the organ donor and the recipient with TB, and the donor’s social ties to patients with outbreak-associated TB. All organ donors in the United States are evaluated to mitigate the risk for infectious disease transmission by organ transplantation. The Organ Procurement and Transplantation Network has defined standards for screening of deceased donors (*3*), and donor evaluation includes a chest radiograph. For lung donors suspected of infection, bronchoscopy specimens for mycobacterial testing including AFB smear and culture are recommended, although addition of NAAT is preferred (*4*). Additional recommendations include ascertaining epidemiologic and medical history from next-of-kin for all deceased solid organ donors to determine TB risk. In this case, the organ donor risk assessment questionnaire administered to family members included questions about TB risk factors, but family members were not aware of or did not recall the donor’s previous positive TST result.

Current guidelines acknowledge the challenges in using epidemiologic data to guide TB risk stratification from deceased donors (*4*). Chemoprophylaxis is recommended only in lung transplant recipients when there is laboratory documentation of untreated or inadequately treated latent TB or apical fibrosis in a donor with epidemiologic risk factors (*4*). It is difficult to evaluate for latent infection in deceased donors with unknown TB status. The 48–72 hour window required to interpret a TST is often incompatible with organ donation, and the test performance of both TST and IGRA might be diminished in persons with brain death (*5*). In this case, the donor’s TST was negative, and the IGRA was indeterminate, at the time of organ donation evaluation. However, TST conversion was documented in records from a previous incarceration, which was unknown to the organ procurement organization at the time of organ recovery.

To prevent future transmissions, if there is clinical suspicion, organ procurement organizations or transplant centers might work with the TB control program in the donor’s state or county of residence to seek further information regarding TB risk (*6*). Even delayed reporting to transplant centers could prevent TB-related morbidity and mortality among recipients through testing and empiric treatment for LTBI. Reporting systems for latent TB are available in some states, and might make this information more readily available (*7*). Organ procurement organizations and transplant centers could also obtain and review medical records from previous medical homes and from high-risk settings such as jails and prisons to ascertain TB risk. However, this might not be feasible for most organ donors.

Genotyping results were crucial to this investigation. The organ donor was epidemiologically linked after death to an ongoing TB outbreak occurring outside the jurisdiction in which the organ recipient resided. Organ recipients and donors are often not located within a single health jurisdiction, and state TB control programs can play a critical role in systematically reviewing and interpreting TB genotypes from organ recipients. This information is essential when determining whether disease was likely to be donor-derived, and this distinction can have significant implications for the recipient with TB and recipients of other organs from the same donor. In addition, analysis of a genotype in an organ recipient might provide additional information, as it did in this case, about the transmission dynamics of associated TB outbreaks. Coordination between organ procurement, transplant center, and public health partners is essential to ensure timely identification of donor-derived TB infection or disease and facilitate prompt clinical interventions to prevent recipient morbidity.

**Summary**

**What is already known about this topic?**

Donor-derived tuberculosis (TB) is a rare but important complication of solid organ transplantation. When donor-derived TB disease is identified in an organ recipient, other patients who received organs from the same donor should be evaluated and, when indicated, treated for TB infection or disease.

**What is added by this report?**

This report describes a case of donor-derived TB disease and illustrates some limitations in determining the TB status of organ donors through medical evaluation or interviewing next-of-kin. This report also describes how the organ recipient’s TB genotyping results helped link the recipient with patients within an ongoing TB outbreak.

**What are the implications for public health practice?**

Clinicians and local health departments (LHDs) should assess the likelihood of donor-derived transmission in organ recipients who develop TB disease. State TB programs can help LHDs obtain and analyze TB genotyping results of organ recipients with TB disease for evidence of possible donor-derived TB. Detection and investigation of suspected donor-derived TB are aided by communication among organ procurement organizations, clinicians, and public health programs at the local, state, and federal level. Retrieving the transplant donor’s medical records from settings such as jails and prisons might augment the predonation TB risk assessment.
